# The Book World of Medicine and Science

**Published:** 1901-02-02

**Authors:** 


### The Book World of Medicine and Science.

The History of Ancient Gynæcology. By W. J. Stewart McKay, M.B., M.Ch., B.Sc., Senior Surgeon to the Lewisham Hospital for Women and Children, Sydney. (London: Baillière, Tindall and Cox. 1901. Price 7s. 6d. net.)

In this work the author has endeavoured to gather together the many fragmentary references to gynæcology found in the medical works of the ancients. Commencing with the Egyptian Papyrus Ebers, the oldest medical work in existence, which dates as far back as 1550 B.C., the Hindu writings dating about 800 and 600 B.C., the medical literature of the Grecian, Roman, and Byzantine periods as late as the opening centuries of the Christian era are briefly outlined.

The book is divided into two parts, the first containing a description of the origin of the various schools of medicine, the lives of the authors, and a short account of their work with reference to gynæcology; the second is a short résumé of the whole "in order that the reader may, if he thinks fit, read the last chapter first." Notwithstanding the fact that the author lives at the Antipodes, and thus had to wait months and even years before some of the works of reference could be obtained, he claims to have neglected no work of any importance among the classical authors containing any passages on gynæcology.

On perusal of this work one cannot help being struck with the extreme antiquity of much of the everyday routine practice of a modern gyncælogist. For instance, vaginal examinations are mentioned in the Papyrus Ebers; in later times Soranus gives advice which is quite applicable at the present day, "the midwife should introduce into the vagina the index finger of the left hand, the nail of which has been evenly pared." The vaginal speculum was used by Soranus 100 B.C., and one has been found during the recent excavations at Pompeii; this instrument was lost sight of during the middle ages until it was reintroduced by Recamiér in 1801. The sound, made of wood or metal, was used to dilate the cervix for the application of drugs, and even to measure the vagina, but not, apparently, as in recent years, as a means of determining the length and position of the uterus. Pessaries and suppositories date from most ancient times. With regard to operations, we find that the Romans were familiar with both the rapid and gradual methods of dilatation of the cervix; at Pompeii seven varieties of uterine dilators have been found, and about this time tents made of sponge or flax were used. The Hindus described paracentesis of the abdomen, warning the operator against injuring a blood vessel or allowing air to enter the abdominal cavity; they also advocated abdominal section for intestinal obstruction, but from the description of most of the operations one cannot help thinking that in many cases more harm than good must have been the result. Yet occasionally the method of procedure was exactly similar to that of the present day; for instance, in opening an abscess of the breast the incision was evidently to be a radiating one, and care was to be taken not to wound the nipple, so as to avoid loss of function. Soranus was aware that traction on the cord produced inversion of the uterus.

We quite agree with the author of the present work in thinking that the history of an important subject such as gynæcology should be written, but such a work ought to have one of two merits: it should either be essentially a work of reference, provided with a complete index (the present work has none, and the cross references in the text are scanty), or it should be an interesting and readable book from a literary and historical standpoint, which we are afraid the work before us cannot claim to be.

It contains much, however, that is useful and interesting, and we cannot help thinking that if in a future edition the author enlarges the *résumé*, giving some space to the developments which have occurred since the opening years of the Christian era, and compiles a full index, he will have produced a book which will fill a useful place in a reference library.

A Prescriber's Companion: Containing Prescriptions, Doses, Weights, and Measures, Hints on Ventilation and Warming, Disinfection, Baths, Prevention of Bedsores, Massage, Electricity, Infant Feeding, and Miscellaneous Information. By Thomas D. Savill, M.D.Lond., D.P.H.Camb., assisted by Agnes F. Blackadder, M.B., Ch.B.Glas., M.A.St.And. (London: John Bale, Sons, and Danielsson. 1900. Price 1s. post free.)

We have no doubt whatever that this little book will be found of constant service by those to whom it appeals. There are two hundred formulæ, which will probably be sufficient for their purpose. In addition to these there are tables of the duration of pregnancy, of the contagious and eruptive diseases, of weights and measures, of saturations, &c., together with a good deal of information of a varied and more or less reliable character. We hardly think, however, that when a man has reached the dignity of being a "prescriber" he is likely to want to carry in his pocket directions how to remove the stains of marking-ink from linen, nor that he ought to have to refer to such a book as this to find out that after cutting operations on the throat the patient should avoid taking crusts, biscuits, or other hard food, or what the temperature of a sick-room ought to be, or that the nurse in charge of an infectious case should wear a blouse overall when on duty. Nor do the details given as to disinfection, bathing, fomenting, and the prevention of bedsores appear such as require to be printed out for the instruction of "prescribers." Can it possibly be that this little work with its ready-made prescriptions is intended for the use of nurses? Surely a medical practitioner would not refer to such a pocket-book to find out on what principle the serum treatment depends, nor ought he to have to appeal to it in regard to the management of the Faradic battery to find out that "unless the battery makes a humming noise it is not in working order," and that "the strength of the current is regulated by pushing the central rod gradually into the coil." We doubt the directions for making beef-tea, and we question whether to "hang a sheet constantly wet with carbolic solution, 1 in 20, across the door or passage" is sufficient to isolate an infectious patient, and there are other points which we might criticise. Still, the book contains a considerable amount of varied information. Our puzzle is to find out for whose use it was written.

What to Do in Cases of Poisoning. By William Murrell, M.D., F.R.C.P. Ninth edition. (London: H. K. Lewis. 1900. Price 3s. 6d.)

To a ninth edition what need one say? In fact all that one has to say about this handy little volume is that it has been well brought up to date, and that the contents of its pages exactly answer the question on its title page—What to do in cases of poisoning? It is a book which ought to lie on the desk of every practitioner, for no man knows at what moment he may not be called upon to treat a case of poisoning; and yet, from the infrequency of such cases, no man's brain can be expected to remember all the required details regarding the vast number of possible poisons. On such a subject the memory requires refreshing, and here is the refreshment, to be taken *pro re nata*—as occasion arises.

